# A short-term evaluation of a prototype disposable Oscillating Positive Expiratory Pressure (OPEP) device in a cohort of children with cystic fibrosis

**DOI:** 10.1186/s12890-021-01525-3

**Published:** 2021-05-12

**Authors:** Kevin J. O’Sullivan, Valerie Power, Barry Linnane, Deirdre McGrath, Magdalena Mulligan, Rebecca White, Leonard W. O’Sullivan, Colum P. Dunne

**Affiliations:** 1 Rapid Innovation Unit - University of Limerick, Limerick, Ireland; 2 School of Medicine, University of Limerick, Limerick, Ireland; 3 University Hospital Limerick, Dooradoyle, Limerick, Ireland; 4 National Children’s Research Centre, Crumlin, Dublin 12, Ireland; 5 Paediatric Cystic Fibrosis Department, University Hospital Limerick, Limerick, Ireland

**Keywords:** Oscillating positive expiratory pressure therapy; Cystic fibrosis; Hypersecretion; Airway clearance; Paediatric

## Abstract

**Background:**

Oscillating Positive Expiratory Pressure (OPEP) devices are important adjuncts to airway clearance therapy in patients with cystic fibrosis (CF). Current devices are typically reusable and require daily, or often more frequent, cleaning to prevent risk of infection by acting as reservoirs of potentially pathogenic organisms. In response, a daily disposable OPEP device, the UL-OPEP, was developed to mitigate the risk of contamination and eliminate the burdensome need for cleaning devices.

**Methods:**

A convenience sample of 36 participants, all current OPEP device users, was recruited from a paediatric CF service. For one month, participants replaced their current OPEP device with a novel daily disposable device. Assessment included pre- and post-intervention lung function by spirometry, as well as Lung Clearance Index. Quality of life was assessed using the Cystic Fibrosis Questionnaire – Revised, while user experience was evaluated with a post-study survey.

**Results:**

31 participants completed the study: 18 males; median age 10 years, range 4–16 years. Lung function (mean difference ± SD, %FEV1 = 1.69 ± 11.93; %FVC = 0.58 ± 10.04; FEV1: FVC = 0.01 ± 0.09), LCI (mean difference ± SD, 0.08 ± 1.13), six-minute walk test, and CFQ-R were unchanged post-intervention. Participant-reported experiences of the device were predominantly positive.

**Conclusions:**

The disposable OPEP device maintained patients’ lung function during short term use (≤ 1 month), and was the subject of positive feedback regarding functionality while reducing the risk of airway contamination associated with ineffective cleaning.

**Registration:**

The study was approved as a Clinical Investigation by the Irish Health Products Regulatory Authority (CRN-2209025-CI0085).

**Supplementary Information:**

The online version contains supplementary material available at 10.1186/s12890-021-01525-3.

## Background

Cystic Fibrosis (CF) is an inherited life-limiting disorder that is characterised by thickened, dehydrated pulmonary secretions, and ineffective mucociliary clearance. As a result, microorganisms are not effectively removed from the airways [1, 2]. This initiates a repetitive cycle of chronic infection, inflammation, and leads typically to bronchiectasis and progressive obstructive airway disease [1]. Airway clearance is therefore a fundamental part of managing patients with CF [2]. The purported benefits of airway clearance were first described in *The Lancet* in 1901 [3], while commercial devices to aid chest physiotherapy in children with CF began to appear in the 1970s [4].

Positive Expiratory Pressure (PEP) therapy was developed to promote mucus clearance by splinting open collapsed airways [5] and by increasing intrathoracic pressure distal to mucus plugging through collateral ventilation via the canals of Lambert and pores of Kohn [6]. PEP therapy is achieved by blowing against a fixed or variable small-exit orifice, which increases pressure by limiting flow. The target “therapeutic range” expiratory pressure is detailed in the literature as 10–20 cm H_2_O [6].

Oscillating Positive Expiratory Pressure (OPEP) therapy was a further development in airway clearance therapy, which has been shown to be at least as effective as traditional chest physiotherapy for mobilising secretions [7, 8]. Oscillating intrapulmonary pressure acts to reduce the viscoelastic properties of the secretions, while short increases in expiratory flow act to mobilise secretions cephalad [6, 9].

Numerous PEP and OPEP devices are commercially available today, with varying levels of associated device-specific complexity, resistance settings, and usability. These devices, however, require regular cleaning to avoid contamination with pathogens exhaled into the device or transferred by contact with the mouthpiece. Contamination of respiratory devices in CF care is well documented [10–12]. Arising thereof there is a heavy burden on patients and their care givers to manage infection control risks. Common CF pathogens include *Staphylococcus aureus*, *Pseudomonas aeruginosa*, *Stenotrophomonas maltophilia* and *Burkholderia cepacia* [13]. A 2017 study by Manor et al. found most airway clearance devices were contaminated after routine use (28/30 devices). After cleaning by the users, only 50% of devices had undergone complete eradication of those microbes, 30% showed partial eradication, and failure was observed in 13% [10]. Cleaning procedures for OPEP devices that are provided by their manufacturers are often divergent from the published guidance of organisations such as the CF Foundation (CFF). For example, some instructions recommend a final step of rinsing the device with tap water, which has been found to be a potential source of pathogenic organisms, whereas the CFF recommends rinsing with sterile water [12, 14]. This is of particular concern in situations where it is not practical or feasible for patients or their care givers to perform these cleaning routines, such as during in-patient antimicrobial treatment.

**Fig. 1:**
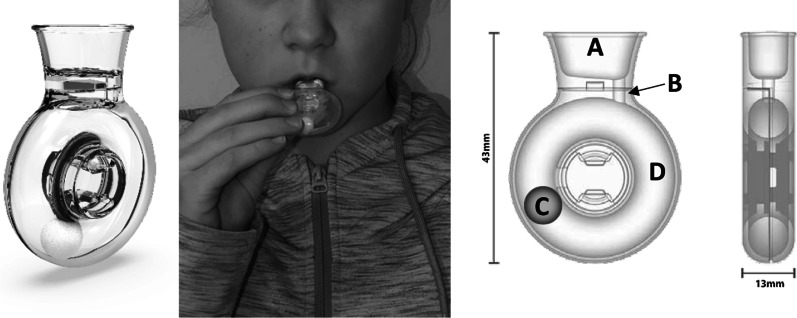
UL-OPEP device

In response to these issues, a novel daily-disposable device was developed in the University of Limerick to eliminate the infection risk associated with contamination and ineffective cleaning (the “UL-OPEP”, Fig. 1—Left). Figure 1—Middle, shows the device in use by a paediatric subject. Figure 1—Right illustrates how the device functions. As the patient exhales into the mouthpiece of the device (A), the fixed orifice (B) restricts expiratory flow to generate a mean increase in intrapulmonary pressure. The expiratory flow exiting the orifice causes the polymer ball (C) to revolve around the annular track (D). This causes the polymer ball (C) to periodically block the orifice, momentarily increasing intrapulmonary pressure to generate oscillations.

The purpose of the current study was to evaluate use of the UL-OPEP device in practice by children with CF over a short-term course of one month, and to determine its effectiveness compared to the current standard of care (i.e., use of existing OPEP devices). As part of this, we also report current device use and cleaning regimens, lung function, Cystic Fibrosis Questionnaire Revised, and usability data for the new disposable device.

## Methods

A cohort of 36 participants was recruited from the paediatric CF service in University Hospital Limerick (UHL) in the mid-west of Ireland, where incidence of CF is highest globally [15]. Potential participants attending regular clinic visits were identified by the clinical staff as current users of at least one hand-held OPEP device and being compliant with OPEP therapy. These potential participants were invited by post to participate in the study, were provided information about the study, and were followed up with a telephone call (two weeks later) to formally complete the recruitment process including consenting formally to participate. Subsequently, they attended scheduled clinics.

A single sample pre-/post-intervention study design was employed, with evaluations and measurements completed in the paediatric CF unit, UHL. All participants were trained by their specialist respiratory physiotherapist to use the UL-OPEP device at the first clinic visit. Each participant was instructed to directly replace their current primary OPEP device with the UL-OPEP device – no change to frequency or duration of therapy was advised. If more than one OPEP device was used regularly by a participant, they were instructed to continue using additional devices as per their current standard of care.

Each participant was then provided with a pack containing 30 UL-OPEP disposable devices (one for each day of the study), instructions for use, and a patient information leaflet.

Specifically, pre- and post-intervention lung function was assessed by a CF specialist physiotherapist using the EasyOne Air Spirometer (NDD Medzintechnik AG, Switzerland) interpreted according to Global Lung Function Initiative 2012 predicted values [16]. Routine measurements of Forced Expiratory Volume in one second (FEV_1_, litres) and Forced Vital Capacity (FVC, litres), FEV_1_ and FVC Predicted (%) [16], and FEV_1_/FVC ratio were collected. Lung Clearance Index (LCI) was assessed via multiple-breath nitrogen washout (Exhalyser D, Eco Medics, Switzerland). A six-minute walk test (6MWT) was performed pre- and post-study as a simple and reproducible test of exercise tolerance for children and adolescents with CF [17]. The Cystic Fibrosis Questionnaire – Revised (CFQ-R) was used to measure health-related quality of life across several domains from the patient’s and/or parent’s perspective [18–20]. The appropriate CFQ-R version was administered according to the age of the participant; CFQ-R Child (6–13 years), and the paired CFQ-R Parent, or CFQ-R Teen/Adult (≥ 14 years). The CFQ-R is scored on a 0–100 scale, with higher scores indicating better health-related quality of life.

A pre-intervention questionnaire recorded the patients’ current OPEP device(s) and associated routines used by each participant, as well as: cleaning methods; cleaning frequency; length of time to clean; and any concerns about cleanliness of reusable devices. User experience of the UL-OPEP device was evaluated with a post-intervention questionnaire, rated on five-point Likert scales (from strongly agree to strongly disagree).

All data were tabulated in Microsoft Excel, and analysed in SPSS Version 26 (IBM, New York, USA). Paired sample t-tests were used to evaluated Pre- and Post- study results.

The study was conducted in compliance with the Good Clinical Practices protocol and the Declaration of Helsinki principle. Approval for this study was granted by the UHL Ethics Board, September 2018. The study was also approved as a Clinical Investigation by the Irish Health Products Regulatory Authority (CRN-2209025-CI0085). Informed consent was obtained from parents/guardians prior to commencement of the study.

## Results

In total 31 participants completed the study: 13 female and 18 male; median age 10 years, range 4–16 years. Of the study cohort, 65% (n = 20) had the homozygous Delta F508 mutation, 29% (n = 9) the heterozygous Delta F508 mutation, and 6% (n = 2) other (G551D/621 + 1G > T, G27X/2622 + 1G > A). Table 1 details summary demographic information regarding participants’ heights and weights as interpreted, in accordance with CDC guidance [21], with data from the Royal College of Paediatrics and Child Health (RCPCH). This is a combination of the Neonatal and Infant Close Monitoring Growth Chart (NICM), the UK WHO 0–4 years growth chart, and the RCPCH UK Growth chart [2–18], and reports lung function as interpreted by the Global Lung Function Initiative 2012 predicted values [16]. Five participants withdrew from the study, one due to unrelated clinical reasons and four due to unrelated personal circumstances.

**Table 1:** Baseline characteristics of the study cohort

	Height			Weight		
	[Cm]	[% Predicted]	[z score]	[Kg]	[% Predicted]	[z score]
Average (SD)	143.4 (17.2)	48.9	−0.035	41.1 (15.0)	61.2	0.425
Minimum	106.0	< 0.4	−3.148	18.2	8.0	−1.404
Maximum	175.0	> 99.6	2.637	76.8	> 99.6	3.310
Median	146.0	48.0	−0.052	39.4	65.0	0.378

	FEV1		FVC		FEV1/FVC	
	[Litres]	[% Predicted]	[Litres]	[% Predicted]	[Ratio]	[% Predicted]
Average (SD)	1.89 (0.85)	79.37	2.41 (1.05)	91.56	0.787	88.91
Minimum	0.7	10	0.96	55	0.52	60.5
Maximum	4.02	114	5.43	121	0.981	109.7
Median	1.6	83	2.14	92	0.804	90

Participants used a variety of PEP/OPEP devices prior to the study. Twenty four used an Aerobika (Trudell, Ontario, Canada), five PEP/RMT (Smiths Medical, NH, USA), five Thera-Pep (Wellspect Healthcare, Molndal, Sweden), and one ‘Bubble Pep’ (i.e. blowing bubbles in a basin). Four used multiple devices. No participants reported using other adjunct therapies such as manual percussion, high-frequency chest wall compression, or intrapulmonary percussive ventilation.

**Fig. 2:**
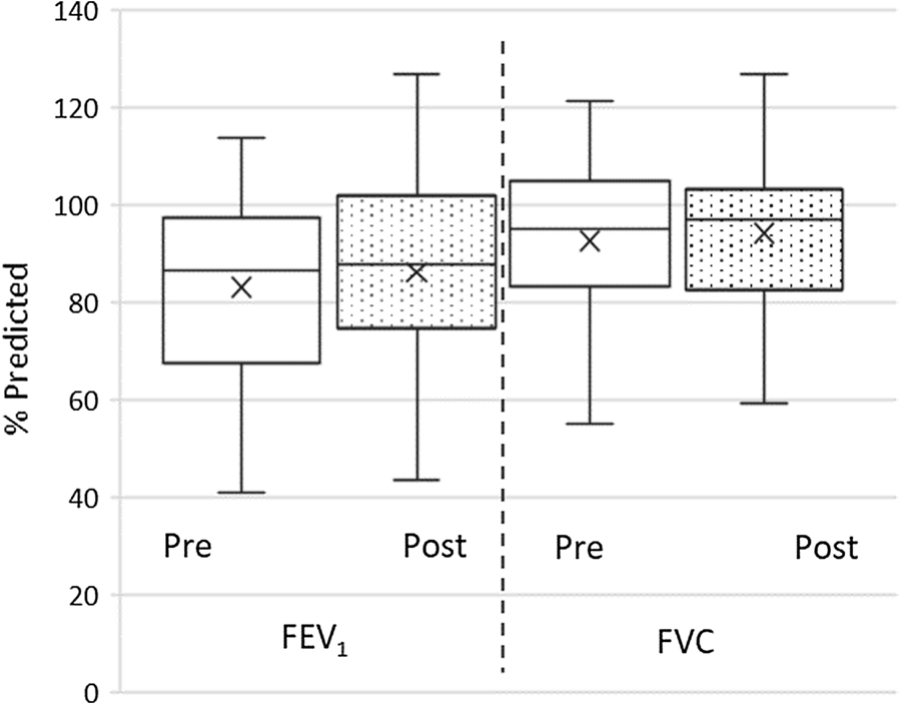
FEV1 and FVC (% predicted) pre- and post-study (Bars = IQR)

**Fig. 3:**
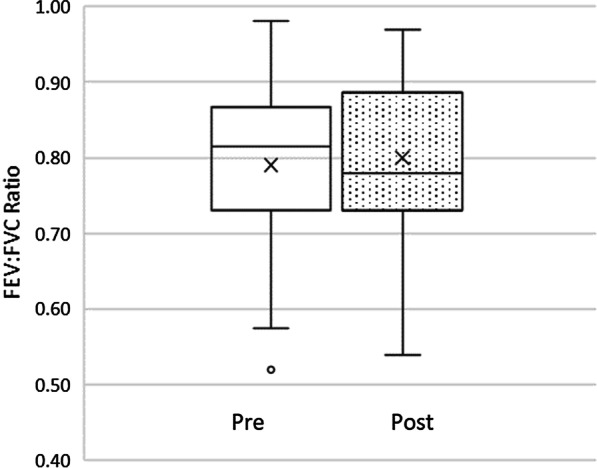
FEV1/FVC Ratio pre- and post-study (Bars = IQR)

**Fig. 4:**
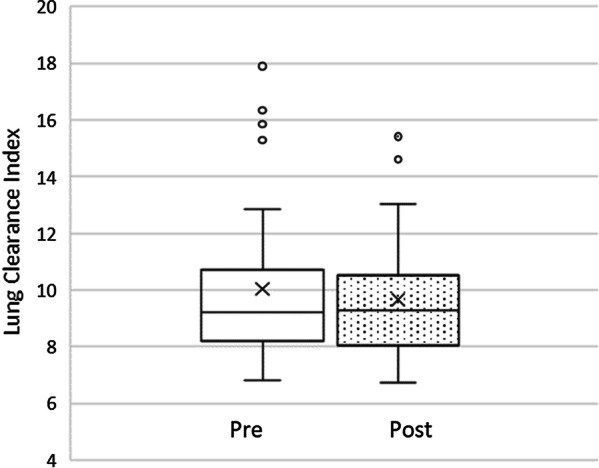
Lung Clearance Index values pre- and post-study (Bars = IQR)

**Fig. 5:**
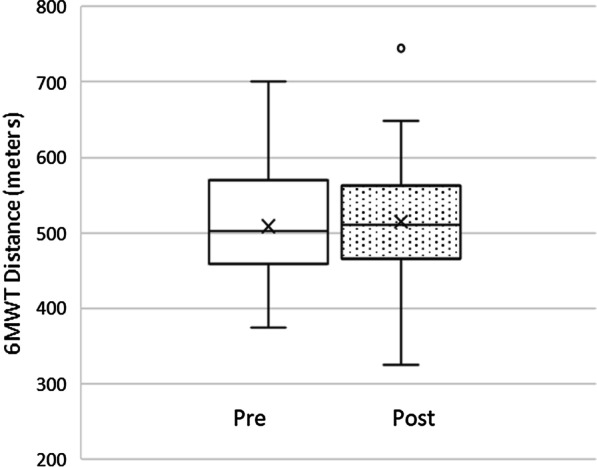
Six Minute Walk Test results (metres) pre- and post-study (Bars = IQR)

There was no significant difference in the scores for FEV1 (Litres) Pre- (x̄ = 1.95, SD = 0.90) and Post- (x̄ = 1.98, SD = 0.87) study; t(29) = −0.840, *p* = 0.408 or, FVC (Litres) Pre- (x̄ = 2.48, SD = 1.12) and Post- (x̄ = 2.52, SD = 1.16) study; t(29) = −0.855, *p* = 0.400 (Fig. 2). FEV1/FVC Ratio Pre- (x̄ = 0.79, SD = 0.11) and Post- (x̄ = 0.79, SD = 0.10) study; t(29) = −0.449, *p* = 0.656 (Fig. 3). Lung Clearance Index Pre- (x̄ = 9.58, SD = 2.29) and Post- (x̄ = 9.66, SD = 2.10) study; t(27) = −0.369, *p* = 0.715 (Fig. 4). 6MWT (meters) Pre- (x̄ = 510.21, SD = 78.59) and Post- (x̄ = 515.03, SD = 86.75) study; t(27) = −0.359, *p* = 0.723 (Fig. 5). 6MWT (SpO2 Avg.) Pre- (x̄ = 96.07, SD = 2.38) and Post- (x̄ = 96.53, SD = 2.78) study; t(27) = −0.826, *p* = 0.416. And, 6MWT (SpO2 1st Minute) Pre- (x̄ = 93.28, SD = 4.25) and Post- (x̄ = 94.39, SD = 4.07) study; t(27) = −1.197, *p* = 0.242. For all box-whisker plots in Figs. 2, 3, 4 and 5, the mean (x), median (horizontal line), interquartile ranges, and outliers (if present) are shown. Table 2 details the frequency of use, duration of use, cleaning frequency, cleaning methods, cleaning duration, and storage habits of the participants.

**Table 2:** Results of usage, cleaning, and storage habits of current OPEP devices

Frequency of use	%
Occasional (only when unwell)	11
Infrequent (2–3 times per week)	14
Once a day	25
Twice a day	44
Three or more times per day	6

Cleaning frequency	%
After each use	53
Daily	17
Every second day	6
Twice a week	5
Weekly	11
Bi-weekly	3
Not at all	5

Duration of cleaning	%
Less than 5 min	15
5–10 min	21
10–15 min	22
15–20 min	15
More than 20 min	27

Duration of use	%
Less than 5 min	6
5–10 min	47
More than 10 min	25
Number of breaths performed	22

Methods of cleaning	%
Hand wash	50
Steriliser	15
Handwash + steriliser	32
Dishwasher	3

Storage habits	%
Open un-protected (countertop/windowsill)	24
Open protected (cupboard/drawer)	18
Sealed container (plastic box/bag)	46
Non-sealed container (soft case)	12

Participants were also asked whether they were ever concerned about the cleanliness of their OPEP devices, to which 24% answered never, 6% rarely, 32% sometimes, and 38% often.

The participant scores at baseline and follow-up for the CFQ-R domains common to both the Child and Teen/Adult versions are presented in Table 3. No significant differences in changes were observed (paired sample t-tests, all *p* > 0.05) in scores pre- and post- study across the dimensions for the participants.

**Table 3:** CFQ-R Pre- and Post-study scores

CFQ-R domain	Baseline, mean (SD)	Follow-up, mean (SD)	Change, mean (SD)
Physical	85.9 (18.6)	84.7 (21.2)	−1.2 (20.4)
Emotion	80.0 (12.4)	80.1 (15.1)	0.1 (8.9)
Social	78.5 (17.0)	79.4 (18.0)	0.9 (13.6)
Body image	79.6 (23.7)	83.3 (14.7)	3.8 (20.0)
Eating	91.1 (15.4)	88.0 (16.6)	−3.1 (13.4)
Treatment burden	81.8 (20.8)	82.2 (18.4)	0.5 (12.6)
Respiratory symptoms	80.1 (17.3)	77.1 (18.9)	−3.0 (18.0)
Digestive symptoms	82.0 (19.9)	74.1 (24.0)	−7.9 (27.9)

**Fig. 6:**
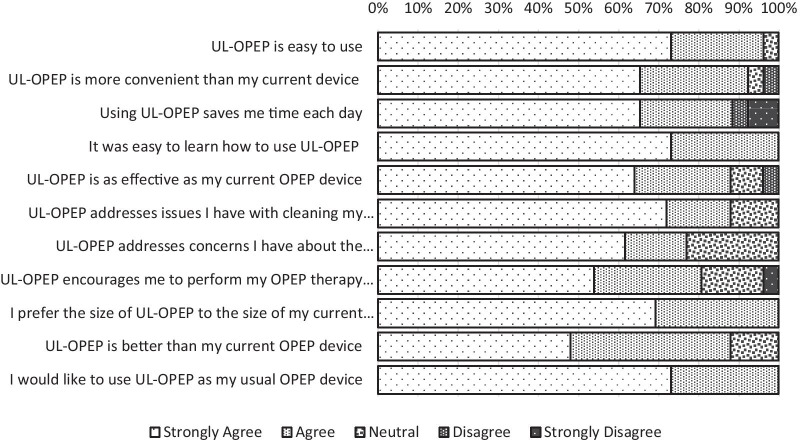
Subjective evluation of usability aspects of the UL OPEP device post study

From the post-intervention device questionnaire, most participants felt that the UL-OPEP device was easy to use (72% Strongly Agree; 24% Agree; 4% Neutral), addressed issues they had with cleaning their current OPEP device (72% Strongly Agree; 16% Agree; 12% Neutral), and encouraged them to perform their OPEP therapy more regularly (52% Strongly Agree; 28% Agree; 16% Neutral, 4% Strongly Disagree). All participants agreed that they would like to use the UL-OPEP as their usual OPEP device (76% Strongly Agree; 24% Agree) (Fig. 6).

## Discussion

This study evaluated the performance of a novel daily use OPEP device over the course of one month in children with CF. In summary, the outcomes indicate that despite its small form, the device performed as well as participants’ existing OPEP devices as an adjunct to airway clearance, with no adverse events recorded during or after the study. There was no deterioration in participants’ health as determined via spirometry, LCI, 6MWT, and health related quality of life measurement. Although the study was of short duration, participants expressed a clear preference for the continued use of the UL-OPEP over their existing device. Notably, there was considerable reduction in time spent dissembling, cleaning, drying, and re-assembling devices daily, and a high level of confidence regarding cleanliness of the UL-OPEP device.

Spirometry results indicate that lung function varied greatly across our sample, yet relative changes in predicted spirometry values observed pre- and post-study were largely within the bounds of normal variability of these measures. For example, up to a 12% relative change in intra-subject FEV_1_ may be considered normal variability for short-term assessment such as the present study [22].

LCI values were higher than previously reported upper limits of normality [23] for 94% of our measurements at baseline, and for 89% at follow-up, despite a large proportion of our participants providing normal or close to normal spirometry results. This may be explained by the fact that LCI is a more sensitive early indicator of peripheral airway disease in children with CF than spirometry [24, 25]. Changes in LCI observed post-intervention are likely due to normal variability, since up to 17–25% variability can be expected in clinically stable school-age children with CF [26, 27].

The 6MWT results indicated that exercise tolerance and functional capacity were unchanged following adoption of the UL-OPEP [28] although, as expected, six-minute walk distance was lower in our sample than reference values for healthy children in the same age range [29].

Changes in all domains within the CFQ-R from pre- to post-study were minimal, and highly variable, indicating that no consistent clinically important changes were observed during the study period. Scores indicate that health-related quality of life across all CFQ-R domains was largely high, but also variable among the sample.

The post-intervention feedback from participants and their parents/guardians regarding the new device was overwhelmingly positive. In particular, all participants agreed that the UL-OPEP was easy to learn to use; preferred the small size of the device to the size of their existing devices; and stated a preference for use the new device as their usual OPEP device, if available to them. The fact that it is both easy to learn how to use initially, and easy & convenient to use generally is of considerable importance since poor performance of OPEP therapy is common among children and adolescents with CF [30].

The results of this study also demonstrate notable variance in frequency of use, duration of use, cleaning regimes and storage of OPEP devices amongst children with CF despite regular standardised education from their specialist physiotherapists. Thus, there is a need to develop devices and related strategies that simplify and reduce the patient/care giver burden associated with airway clearance therapy.

## Limitations

This is the first study evaluating this prototype disposable OPEP device. While acknowledging the limited sample size in the study, the study design was based on previous registered clinical trials where short term studies of OPEP devices were conducted for period of up to four weeks [31, 32]. In the context of this relatively brief duration, results are not over-stated and the conclusions drawn are modest, albeit promising. A larger, longer duration study would be required to enable generalisation of results to the wider CF patient population.

It is acknowledged that long-term use of a daily disposable OPEP device would most likely increase costs compared to current offerings. The UL-OPEP is not currently commercially available and so actual cost-in-use figures, which would be based on large-scale manufacture and distribution models, are unknown. However, it is reasonable to speculate that the cost of routine or long-term daily use may limit accessibility unless supported by local health care systems or health insurance. Despite this, the potential benefits of employing a cost-effective disposable device for short term specific-purpose use such as travel, clinic evaluations, and in-patient stays are attractive.

## Conclusions

Our findings demonstrate that the novel daily disposable device has equivalent performance to currently available OPEP devices in practice, at least over a relatively short period of one month, with no risk of contamination or infection due to ineffective cleaning regimes that are no longer required. Participants reported a preference for the new device over their existing OPEP devices due to ease of use, convenience, and efficacy it provided. Therefore, it may be a useful adjunct to aid airway clearance in children with CF and may promote greater adherence and compliance to airway clearance.

In the context of the current coronavirus pandemic, a disposable respiratory device appears both relevant and prudent, especially for patient with respiratory conditions or susceptibility to infection for whom hospital stays may be relatively frequent and unavoidable.

## Supplementary Information

**Additional file 1**. Post-Study Questionnaire.

## Data Availability

The datasets used and/or analysed during the current study are available from the corresponding author on reasonable request.
