# Supplementary material for: A short-term evaluation of a prototype disposable Oscillating Positive Expiratory Pressure (OPEP) device in a cohort of children with cystic fibrosis
Source: BMC Pulm Med. 2021 May 12;21:158. doi: 10.1186/s12890-021-01525-3 (PMC8114193; doi:10.1186/s12890-021-01525-3)
Supplement: Supplementary file 1 — Additional file 1. Post-Study Questionnaire. [file 12890_2021_1525_MOESM1_ESM.docx]

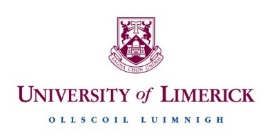

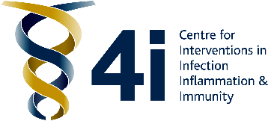


| ID: ____________ | Date: ____________ |
| --- | --- |

**Post-Study UL-OPEP Questionnaire**

**Please tick the box that best describes how you feel about the UL-OPEP Device.**

|  | Strongly Agree | Agree | Neutral | Disagree | Strongly Disagree |
| --- | --- | --- | --- | --- | --- |
| UL-OPEP is easy to use |  |  |  |  |  |
| UL-OPEP is more convenient than my current device |  |  |  |  |  |
| Using UL-OPEP saves me time |  |  |  |  |  |
| It was easy to learn how to use UL-OPEP |  |  |  |  |  |
| UL-OPEP is as effective as my current OPEP device |  |  |  |  |  |
| UL-OPEP addresses issues I have with cleaning my current device |  |  |  |  |  |
| UL-OPEP addresses concerns I have about the hygiene of my current device |  |  |  |  |  |
| UL-OPEP would encourage me to perform OPEP therapy regularly |  |  |  |  |  |
| I prefer the size of UL-OPEP to the size of my current OPEP device |  |  |  |  |  |
| I would like to use UL-OPEP as my usual OPEP device |  |  |  |  |  |
